# Exploring psychological dimensions of augmented reality in education: a study on learning motivation and achievement in museums

**DOI:** 10.3389/fpsyg.2025.1514117

**Published:** 2025-01-29

**Authors:** Anping Cheng, Weiru Zhang, Anran Feng, Yunmengru Wu, Wanjun Li

**Affiliations:** Birmingham Institute of Fashion and Creative Art, Wuhan Textile University, Wuhan, China

**Keywords:** augmented reality, educational psychology, learning motivation, academic achievement, museum learning

## Abstract

Augmented reality (AR) has gained significant attention and is being increasingly utilized to enrich the learning experience of museum visitors. This study explores the psychological dimensions of AR in education, focusing on learning motivation and academic achievement in museums. A quantitative research study was carried out, encompassing a survey of 266 visitors at the Wuhan Natural History Museum. Partial Least Squares Structural Equation Modeling (PLS-SEM) was employed as the analytical tool to validate the proposed model. The findings reveal significant positive effects of information quality on immersion, imagination, and academic achievement, as well as positive effects of information richness on academic achievement. Information quality mediates the relationship between information richness and immersion/imagination, while immersion and imagination mediate the association between information quality and learning motivation. Additionally, learning motivation positively influences academic achievement. Furthermore, wearable comfort moderates the effect of information quality on immersion and imagination. The study provides theoretical insights into the complex interplay between these variables and their impact on learning motivation and academic achievement. The findings have implications for the design of AR-based learning systems and highlight the importance of considering wearable comfort in enhancing user experiences.

## 1 Introduction

According to the ICOM 2022 definition, a museum is a non-profit, permanent institution that serves society by researching, collecting, conserving, and exhibiting tangible and intangible heritage. Open to the public, museums promote inclusivity, diversity, and sustainability while fostering education, enjoyment, and knowledge sharing through community participation. Since the 19th century, museums have been acknowledged by educators as invaluable informal learning spaces, offering the general public, ranging from children to adults, the opportunity to access visual, tangible, and accessible exhibits to explore and deepen their understanding of art, history, culture, and science. Furthermore, museums also play a crucial role in developing children’s interests and shaping their career aspirations in the fields of art, history, culture, and science (Kisiel, 2006; De Rijcke and Beaulieu, 2011; Lewalter et al., 2021). Museums are recognized for featuring exhibits, collections, and experiences meticulously designed to fulfill educational targets aimed at school-aged children, as pointed out by Crowley et al. (2014). Museum educators frequently confront the challenge of developing captivating and interactive learning activities for visitors (Watson and Werb, 2013; Zhou et al., 2022).

In the contemporary era of technological advancement, the education system is diversifying rapidly. Novel technologies are readily accessible and incorporated into e-learning systems, serving as innovative cognitive tools that facilitate communication and interaction between students and teachers (Fidan and Tuncel, 2019; İbili et al., 2020; Sirakaya and Alsancak Sirakaya, 2018; Turkan et al., 2017). In addition, the use of visualization on smart devices has the potential to have a major impact on various learning contexts by addressing the limitations of traditional learning systems. Augmented reality (AR) and virtual reality (VR) technologies have surfaced as cutting-edge tools, increasingly incorporated into museum educational programs, aimed at crafting more captivating and immersive learning journeys for visitors (Crowley et al., 2014). Unlike VR, which fully submerges users in a simulated realm, AR merges virtual elements with physical surroundings (Carmigniani et al., 2011; Garzón et al., 2019). This integration is achieved by superimposing real-world objects or their digitized counterparts with supplementary textual, audio, video, or other virtual content, referred to as “digital augmentations” (Sommerauer and Müller, 2014). The effect of superimposing digital augmentations is often achieved through the use of mobile platforms, where a camera view is used to overlay the virtual elements onto real-world objects. Smart glasses with see-through displays can also be used to achieve this effect (Pellas et al., 2019; Scavarelli et al., 2021).

Numerous empirical studies have explored the utilization of AR technologies in museum-based education across diverse fields such as science, art, archeology, medicine, and the military, among others (Zhou et al., 2022). AR can enhance learners’ access to phenomena that were previously inaccessible or unseen, as well as digital information pertaining to exhibits, ultimately fostering a deeper understanding of the real world (Ibáñez and Delgado-Kloos, 2018; Danaei et al., 2020). Oh et al. (2017) has introduced a collaborative, multiuser simulation titled ARfract. This innovative simulation leverages cutting-edge technologies, including optical see-through AR glasses, projection-based AR, and gesture recognition, to immerse visitors in an interactive learning experience that delves into complex concepts such as the refraction of light. Pasaréti et al. (2011) posited that AR has the capability to comprehensively showcase the distinctive features of virtual representations of objects within a genuine real-world setting. Saleem et al. (2021) findings suggest that students exhibit a profound preference for e-learning via an augmented reality application. The dissemination of information via an augmented reality application favorably influences attitudes, subjective norms, and perceived behavioral control. According to Yoon et al. (2017), even with limited time for exploration in a science museum, people who utilized AR technology demonstrated greater gains in knowledge compared to those who did not use it. Several studies have indicated that the utilization of AR in museum education can enhance learners’ thinking skills, including creative thinking (Guazzaroni, 2013; Hammady et al., 2020), inquiry (Hsiao et al., 2016), and critical thinking skills (Poce et al., 2019). AR glasses enhance visitor experiences with features like first-person view and context-aware information. Studies show AR glasses outperform smartphones in delivering immersive and interactive learning in cultural heritage sites, with strong user acceptance despite some usability challenges (Litvak and Kuflik, 2020). AR glasses outperform tablets in enhancing learning effectiveness and motivation in museum-based language learning, offering a more immersive and interactive experience. Their integration also highlights the influence of learning styles, with kinesthetic learners benefiting most from AR glasses strategies (Chen et al., 2023). AR glasses usage in heritage museums enhances user satisfaction and influences post-experience intentions, including continued AR use and destination revisits. Factors such as technical novelty, trust, and situational aesthetics highlight AR glasses’ potential for immersive and educational tourism (Chen et al., 2024). Existing literature on the application of AR in informal learning environments highlights the pivotal role of psychological factors, such as immersion, imagination, and motivational constructs, in enhancing user engagement and learning outcomes (Huang et al., 2021; Kim and Lee, 2021). However, most studies have overlooked how key variables—such as wearable comfort, immersion, and imagination—interact to influence learning motivation and academic achievement in museum contexts. To address this gap, our study investigates how wearable comfort moderates the effect of information quality on immersion and imagination, and how these factors contribute to learning motivation and academic achievement in AR-based museum learning.

Based on the literature review, we established research dimensions and proposed research hypotheses in Section 2. In Section 3, we designed a questionnaire based on existing references. Subsequently, participants were recruited to explore museum exhibits utilizing AR equipment, and subsequent data was collected. In Section 4, we analyzed the data and presented the verification of the research hypotheses. Finally, Section 5 provided a summary of the research results and discussion, along with future research directions and suggestions.

## 2 Literature review and hypotheses development

### 2.1 Wearable AR technology in museums

Augmented reality (AR) is widely recognized as a highly promising digital technology for enhancing the museum visitor experience (Zhuang et al., 2021; Li et al., 2024). High-quality AR fosters immersive experiences that, in turn, enhance users’ perceptions of usefulness and ease of use. Aesthetic immersion also promotes escapist immersion, offering valuable insights for effective AR design in museums (Cheng et al., 2024).Memorable tourism experiences significantly boost place attachment through hedonic and eudaimonic wellbeing, underscoring the importance of positive psychological factors in fostering loyalty and revisit intentions (Vada et al., 2019). High-quality AR in museums significantly enhances both utilitarian and hedonic value, leading to more engaging tour experiences and improved psychological wellbeing. Content quality, system quality, and vividness are key factors driving AR adoption, highlighting the importance of well-designed AR for enriching visitors’ mental health and overall museum experience (Ma et al., 2024).

### 2.2 Information quality and information richness

In this study, the selection of variables was primarily guided by the Information Systems Success Model (DeLone and McLean, 2003), which highlights the importance of information quality and its downstream effects on user behavior and satisfaction. Given the study’s objective to examine psychological dimensions and their impact on learning motivation and academic achievement, this model was deemed more suitable. While other well-established frameworks, such as the Technology Acceptance Model (Venkatesh and Bala, 2008) and the Unified Theory of Acceptance and Use of Technology (Venkatesh et al., 2012; Tamilmani et al., 2021), include constructs like perceived ease of use, usefulness, enjoyment, and self-efficacy, these models primarily focus on technology adoption and initial acceptance, making them less aligned with the current study’s goals. A study has revealed that user satisfaction with Instagram is influenced by two primary factors: the strength of word-of-mouth recommendations and the quality of the information shared on the platform (Danniswara et al., 2020). Zha et al. (2018) found that focused immersion plays a positive moderating role in the relationship between information quality and informational fit-to-task. Kim and Lee (2021) study revealed a significant influence of online class quality on immersion. Moreover, they found a positive relationship between online class quality and learning satisfaction. An et al. (2021) discovered that two essential characteristics of virtual travel, namely the sense of immersion and the quality of information, were identified and shown to exert a beneficial influence on tourists’ flow experience.

The information richness theory, alternatively referred to as the media richness theory (Daft and Lengel, 1986), conceptualizes communication channels as objective characteristics that dictate their capacity for information transmission. This theory encompasses four essential attributes, including the promptness of feedback, the capacity to convey multiple cues, linguistic diversity, and the likelihood of personal attention. Face-to-face communication is considered richer in information as it is bidirectional and can help alleviate comprehension challenges. However, in the context of museum studies, communication typically involves one-way persuasive communication. Chen and Chang (2018) suggested that websites have evolved significantly in terms of media richness, evolving from static textual and graphical elements to dynamic and interactive virtual experiences. This evolution aims to enhance satisfaction and confidence. Chesney et al. (2017) research revealed that the information richness offered by the virtual world interface has the potential to enhance e-commerce transactions by cultivating trust between trading partners. Jiang et al. (2022) utilized a structural equation model to explore the determinants of users’ continuance intention with museum AR technology and uncovered that information richness exerts a favorable impact on information quality.

AR has been shown to be an effective tool for presenting various museum collections, including painting guides (Ding, 2017), narrative interactions, stories behind statues (Keil et al., 2013), and learning experiences of antique relics (Khan et al., 2021). Research conducted in the field of AR applications in art museums indicates that these technologies offer visitors a more profound understanding of the intricate details and wealth of information contained in paintings, surpassing the capabilities of traditional guides, by offering more information and better information quality (tom Dieck et al., 2018). Visitors equipped with AR guides exhibit increased focus and engagement with museum artworks, ultimately leading to enhanced learning efficiency and a more immersive flow experience (Chang et al., 2014). Visitors have expressed a desire for AR devices to provide high-quality information and better learning experiences (Jiang et al., 2022).

Additionally, several researchers have examined the relationship between information quality and information richness. Aljukhadar and Senecal (2017) uncovered that the perceived information quality among participants who had recreational browsing intentions was significantly influenced when product details on an e-commerce website were presented through streaming video. Zhao et al. (2017) indicated that the rich media on websites had a positive impact on consumers’ perception of information quality. Jiang et al. (2022) undertook a study examining the elements that shape users’ intention to continue utilizing AR technology in museums, revealing that information richness positively contributes to information quality. Therefore, this study proposes the hypothesis that:

H1. Information quality has positive effects on immersion.

H2. Information quality has positive effects on imagination.

H3. Information quality has positive effects on academic achievement.

H4. Information richness has positive effects on academic achievement.

H5. Information richness has positive effects on information quality.

### 2.3 Immersion and imagination

Immersion can be perceived in different ways, either as an aspect of the technology itself or as a physical or psychological reaction. It can be a bodily sensation, similar to being immersed in water, or a mental state, such as being absorbed in a good book (Witmer and Singer, 1998). Museums aim to provide immersive and memorable participatory experiences to visitors (Vesci et al., 2020). The modern museology highlights the significance of fostering visitors’ active participation and engagement, moving beyond mere displays of valuable cultural artifacts (Savenije and De Bruijn, 2017). Immersive experiences are crucial in enhancing the overall satisfaction (Scholz and Smith, 2016). Kim and Lee (2021) pinpointed the challenges of online education from the viewpoint of learners and observed a positive correlation between immersion and academic achievement. Georgiou and Kyza (2018) investigated the possible effect of student motivation on the impact of immersion in learning environments utilizing location-based AR technologies. Their study revealed a positive relationship between the level of immersion and conceptual learning gains. Huang et al. (2021) conducted a study to investigate how learners’ motivation, engagement, performance, and spatial reasoning evolve over time, taking into account varying degrees of immersion. The findings indicated that higher levels of immersion were associated with increased levels of motivation and engagement among learners. Cheng and Tsai (2020) confirmed that students’ perceived immersion in a virtual learning environment is associated with their learning perceptions and serves as a predictor of positive affective outcomes.

Burdea and Coiffet (2003) define imagination as a characteristic elicited by the content of virtual environment applications, wherein the user’s mind possesses the ability to perceive non-existent entities or concepts. Madini and Alshaikhi (2017) discovered that the variable of imagination held significant importance for high-immersion VR systems that utilized head-mounted displays. Huang et al. (2010) found that virtual reality learning environments have the capability to simulate the real world, thereby stimulating the learner’s imagination and enhancing their capacity for conceptualization. Creative imagination grants learners the ability to mentally envision novel ideas and concepts that are not immediately discernible through their senses (Singer, 2000), thereby facilitating users in accomplishing their learning goals (Huang et al., 2010). Burdea and Coiffet (2003) argue that the assessment of user attitudes and receptiveness toward learning system acceptance should incorporate the factor of imagination. Huang et al. (2010) study revealed that virtual learning environments that stimulate imagination serve as effective tools for enhancing learners’ problem-solving abilities. Huang et al. (2016) performed a research investigating learners’ acceptance of desktop and projection-based display systems in the context of medical education. The research revealed that imagination had a positive impact on learners’ perception of the usefulness and ease of use of virtual learning environments, ultimately influencing their behavioral intention to adopt such systems. Therefore, this study proposes the hypothesis that:

H6. Immersion has positive effects on learning motivation.

H7. Imagination has positive effects on learning motivation.

### 2.4 Learning motivation and academic achievement

Several researchers have highlighted the significance of academic achievement as an essential construct, emphasizing its role as an educational outcome (Choi and Kim, 2013). Academic achievement is commonly defined as the extent to which students accomplish educational objectives. It typically encompasses attaining specific outcomes in online assignments and assessments, often quantified as a grade point average (GPA) or a numerical ranking (Chowa et al., 2015). Two key approaches prevail in the study of academic achievement. Firstly, the quantitative approach relies on the assessment of students’ grade point average (GPA). Alternatively, an alternative method employs a knowledge acquisition and achievement framework to uncover abstract influencing factors (Choi and Kim, 2013). According to some studies, it has been suggested that students, especially those in lower grades, may tend to overestimate their own performance in a social expectation report, which can potentially impact the validity of the findings. In future research, it is recommended to utilize actual online results of students instead of relying solely on the online scores reported by the students themselves. This approach would help eliminate potential social biases (Broadbent and Poon, 2015).

Prior studies have investigated the relationship between learning motivation—comprising of both internal and external motivations—and GPA, which is used as an indicator of academic performance (Choi and Kim, 2013). A cross-lagged regression model was employed to analyze the interrelationships between academic achievement and motivation among high school students. The findings revealed that autonomic motivation, which measures the level of relative autonomy over the past year, exhibited a positive correlation with academic achievement even after accounting for baseline achievement (Taylor et al., 2014). This discovery underscores the crucial role of learning motivation as a key predictor of academic achievement. The research indicates that students who are deeply engaged in the joy and challenges presented by learning motivation are more likely to attain greater academic achievements (Choi and Kim, 2013). Su and Cheng (2019) investigated the impact of a virtual chemistry laboratory, utilizing the sustainable innovation experiential learning model, on academic achievement. The research outcomes demonstrated a favorable correlation between learning motivation and academic achievement, as revealed through survey analysis. Therefore, this study proposes the hypothesis that:

H8. Learning motivation has positive effects on academic achievement.

### 2.5 Wearable comfort

The term “wearable comfort” is defined as the overall subjective assessment of the physical sensation experienced by consumers when wearing AR glasses (Knight and Baber, 2005). Kuru and Erbuğ (2013) introduced the construct of “wearability,” which refers to the specific characteristics of a device, including its ease of carrying and how well it fits with the human anatomy. Kress et al. (2020) argues that two fundamental factors that define the major challenges in mixed reality hardware are comfort and immersion. In their qualitative study of Google Glass, tom Dieck et al. (2016) observed that users frequently commented on the weight of the devices. In the context of AR and VR Smart Glasses, Rauschnabel (2018) posits that the comfort of wearable devices is influenced by a range of their physical attributes, including factors such as weight, bulkiness, operating temperature, and physical pressure experienced while wearing the glasses. Herz and Rauschnabel (2019) formulated and evaluated a comprehensive framework to analyze consumer reactions toward wearable VR glasses. The study revealed that wearable comfort positively influences the attitude toward using VR glasses. Consistent with previous research, we suggest that utilizing AR glasses that provide a high level of wearable comfort results in a positive user experience. Therefore, this study proposes the hypothesis that:

H9. Wearable comfort positively moderates the relationship between information quality and immersion.

H10. Wearable comfort positively moderates the relationship between information quality and imagination.

## 3 Methodology

### 3.1 Study context

**Figure 1:**
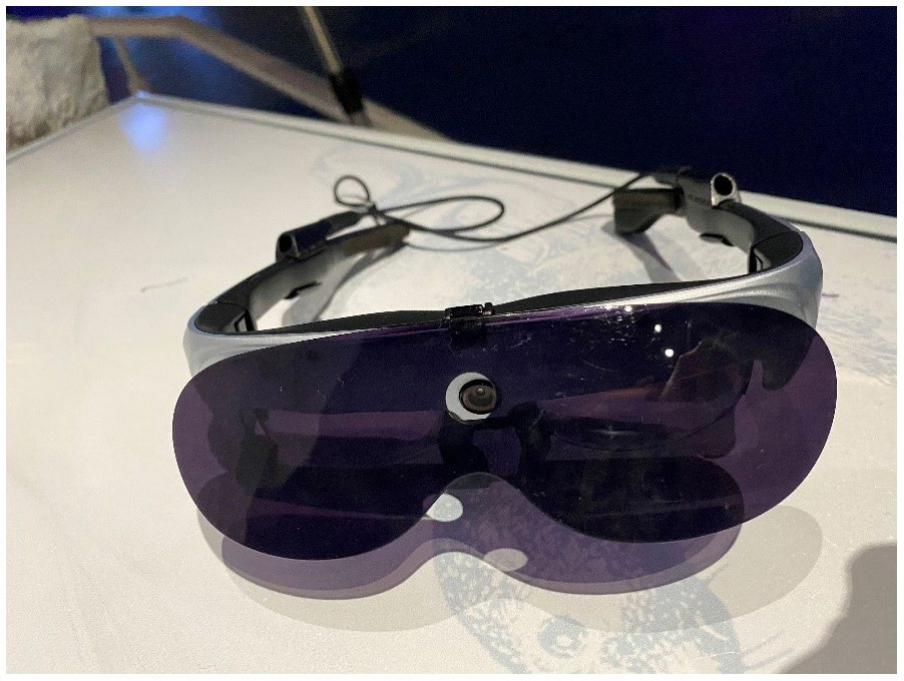
Rokid air AR glasses.

**Figure 2:**
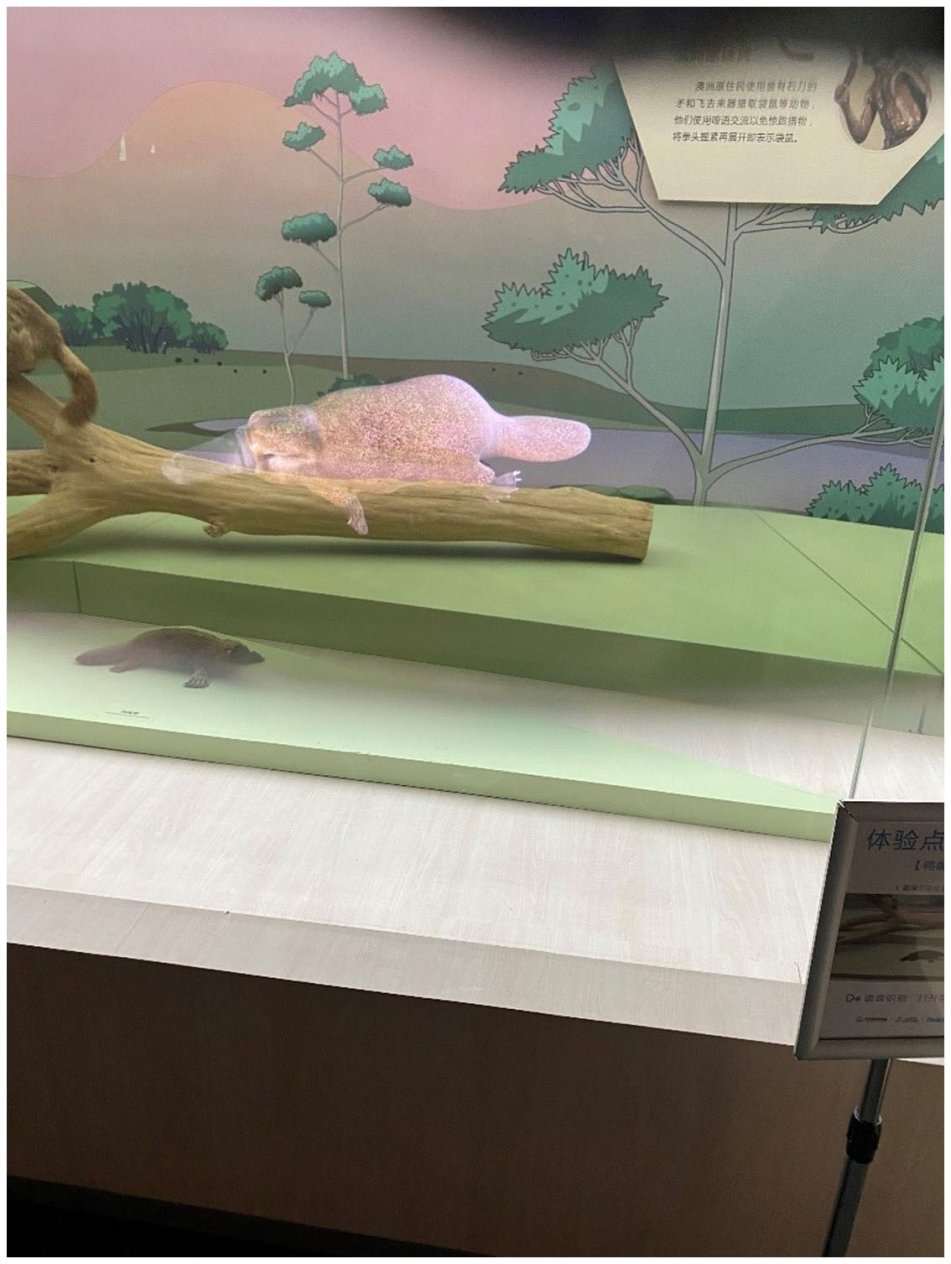
An example of AR-based museum exhibitions.

The Wuhan Natural History Museum is devoted to amassing a diverse array of natural specimens, encompassing animals, plants, artifacts from the Paleolithic era, and fossils pertaining to paleoanthropology. It serves the purpose of educating the public, hosting exhibitions, and disseminating scientific knowledge. To enhance the visitors’ overall viewing experience, the museum has embraced the use of AR as a new medium. Specialized AR glasses, such as the Rokid Air, are provided to tourists to enrich their viewing experience, as shown in Figure 1, allowing them to access AR animations. The Rokid Air AR smart glasses were chosen for their lightweight design (83 g), affordability, and practical features, including a 1080p OLED display and 43° field of view. Compared to higher-cost enterprise-focused models like Magic Leap 2, Rokid Air offers an accessible and suitable option for museum applications. During their museum visits, tourists wear these AR glasses, and the AR content is activated by positional markers positioned in front of each exhibit, which are identified by corresponding signposts. The AR content included 3D animations, visual overlays, and auditory narratives, such as prehistoric creatures in motion or reconstructions of Paleolithic artifacts. Through the AR glasses, visitors gain access to visual and auditory information, seamlessly integrating traditional exhibits with cutting-edge technology, as depicted in Figure 2.

### 3.2 Survey design

Face-to-face surveys were conducted with visitors who interacted with the AR-integrated exhibits at the Wuhan Natural History Museum. Of the 297 visitors who utilized the AR technology and completed the survey, 31 responses were excluded due to incomplete data. The minimum sample size necessary for this research was established using G*Power software to guarantee the statistical significance of the findings (Faul et al., 2009). With a medium effect size of 0.15, a desired statistical power of 0.80, and an alpha level set at 0.05, the sample size calculation considered a model incorporating a maximum of 7 predictors. Based on the calculations, a minimum of 55 respondents was necessary for the experiment. The sample size of 266 participating tourists was deemed sufficient for data analysis. Descriptive statistics of the participants can be found in Table 1.

**Table 1:** Sample characteristics.

Characteristics	Frequency (*n* = 266)	Percentage (%)
Gender		
Male	142	53.38%
Female	124	46.62%
Age		
18–25 years old	115	43.23%
25–35 years old	109	40.98%
over 35 years old	42	15.79%
Occupation		
Student	109	40.98%
Employed	114	42.86%
Unemployed	26	9.77%
Retired	17	6.39%
Familiarity with AR		
Never use	166	62.41%
Rarely use	82	30.83%
Often use	18	6.77%

The survey consists of two sections. The initial section assesses respondents’ level of familiarity with AR, while the subsequent section comprises inquiries relating to seven distinct measurement constructs: information quality, information richness, immersion, imagination, learning motivation, academic achievement, and wearable comfort. All measurement constructs in this study were evaluated using a multi-item 7-point Likert scale, spanning from 1 (strongly disagree) to 7 (strongly agree). The measurement constructs employed in this study were tailored from previous research, incorporating minor adjustments in wording to achieve greater congruence with the precise emphasis on AR as the subject of this research.

### 3.3 Procedure

Participants were approached on-site at the Wuhan Natural History Museum as they expressed interest in experiencing the AR exhibits, and participation was entirely voluntary. On average, visitors wore the AR glasses for about 20–30 min; only a few reported mild discomforts, which dissipated quickly upon removing the glasses. Immediately after using the AR glasses, participants were guided to a quiet seating area within the museum to complete an electronic questionnaire, which typically took around 5–10 min. This immediate post-experience survey helped ensure that responses accurately reflected visitors’ recent interactions with the AR technology. Data collection spanned 4 weeks, during which we maintained a consistent procedure for recruiting volunteers and administering the survey, thus ensuring methodological rigor throughout the study.

### 3.4 Measurement

The entirety of the measurement items utilized in this study were derived from previous research. Specifically, three items measuring information quality were adapted from studies conducted by Roca et al. (2006), while three items assessing information richness were adapted from studies conducted by Oh et al. (2009) and Otondo et al. (2008). Additionally, three items of immersion and three items of imagination were adapted from Barrett et al. (2021) and Bueno et al. (2020). Four items pertaining to learning motivation were adapted from Pintrich and Schrauben (1992), while four items related to academic achievement were adapted from Peterson et al. (2010). Three items of wearable comfort were adapted from studies conducted by Herz and Rauschnabel (2019) and Sukwadi et al. (2022). Ahead of administering the survey to the participants, a pilot test was executed with university students in China. Additionally, following the revisions, five researchers scrutinized the measurement items. A detailed compilation of these items is presented in Table 2.

**Table 2:** Descriptive statistics and factor loading.

Construct/Item	Mean	STD	Loading	*t*-value
Information quality				
IQ1:The information and content provided by the device’s augmented features are easy to understand.	5.49	1.03	0.91	62.30
IQ2: The device’s augmented reality features provide clear information and content.	5.52	0.97	0.92	79.33
IQ3: The device’s augmented reality features present information in the form of an appropriate interface.	5.59	1.01	0.92	80.49
Information richness				
IR1: The device’s augmented reality features can deliver information in multiple ways.	5.56	1.09	0.86	45.38
IR2: The augmented reality features of the device allow me to understand the symbolic meaning of the exhibits in addition to displaying them.	5.50	1.11	0.83	35.47
IR3: Overall, the device’s augmented reality features provide me with a wealth of information about exhibits.	5.78	1.21	0.85	49.19
Immersion				
IMRN1: I feel immersed in the AR environment.	5.44	1.17	0.88	42.57
IMRN2: I feel fully engaged by the AR learning environment.	5.53	1.15	0.94	94.50
IMRN3: I feel like I am in the AR learning environment.	5.52	1.13	0.92	69.39
Imagination				
IMGN1: The use of AR allows me to experience things I do not experience in daily life.	5.61	1.19	0.91	80.15
IMGN2: The use of AR enhances my comprehension of the exhibits.	5.52	1.20	0.86	43.19
IMGN3: The use of AR allows me to immerse myself in the museum environment	5.71	1.12	0.90	63.20
Learning motivation				
LM1: I am intrigued by the initial implementation of AR in museums.	5.46	1.40	0.90	73.11
LM2: I am really interested in the content of the AR.	5.78	1.16	0.90	102.40
LM3: When I learn through using the AR in museums, I am confident to comprehend the exhibits.	5.57	1.28	0.91	104.22
LM4: I derive great pleasure from the AR experiences in museums, which further fuels my desire to expand my knowledge and understanding.	5.69	1.18	0.89	78.66
Academic achievement				
AA1: Learning teaches me something I did not know before.	5.58	1.07	0.83	34.10
AA2: I learn a lot through participating in various AR content.	5.60	1.11	0.86	39.79
AA3: Learning makes me more independent and confident.	5.72	1.13	0.82	29.52
AA4: When I can explain things to others, I know what I have learned	5.57	1.04	0.72	20.28
Wearable comfort				
WC1: Wearing AR glasses is comfortable	5.58	1.04	0.84	30.99
WC2: It feels good to wear AR glasses	5.50	1.00	0.83	31.73
WC3: Using the AR device is convenient	5.88	1.03	0.86	34.83

### 3.5 Data analysis

The model underwent testing with the utilization of SmartPLS 4. According to Hair et al. (2011), Partial Least Squares Structural Equation Modeling (PLS-SEM) is a suitable method for predicting complex models that involve numerous structures, indicator variables, and structural paths, without requiring assumptions about the distribution of the data. PLS-SEM is advantageous in that it does not rely on normality assumptions and can effectively handle small sample sizes (Hair et al., 2019). Moreover, PLS-SEM is compatible with resampling methods, which are considered more effective than traditional tests like the Sobel test, particularly for analyzing indirect effects. PLS-SEM operates through two primary stages: the measurement model and the structural model. The measurement model evaluates the relationships between latent variables and their observed indicators, ensuring reliability and validity. The structural model assesses the relationships between latent variables, testing the proposed hypotheses. Additionally, PLS-SEM supports advanced features like bootstrapping and permutation tests to analyze indirect effects and enhance statistical rigor. Given its ability to handle complex models with numerous constructs and paths, PLS-SEM is widely adopted in various fields, including social sciences, marketing, and information systems. Its flexibility and focus on prediction make it a robust tool for addressing intricate research questions and providing actionable insights.

### 3.6 Common method variance

Since all the data originated from a single source, there is a potential concern regarding common method variance (CMV). In PLS-SEM, two commonly used approaches are employed to assess CMV. The initial methodology involves a thorough collinearity evaluation employing variance inflation factors (VIFs) (Kock, 2015). Following the recommendations of Kock (2015) and Kock and Lynn (2012), all variables should be regressed against a common variable. If the resulting VIF value is 3.3 or below, it signifies the absence of bias in single-source data. The satisfactory VIF values for all constructs indicate that the presence of CMV was not a significant concern in this study.

The second approach for assessing CMV employs the correlation matrix procedure. Under this approach, if the correlations among constructs remain under 0.9, it signals the absence of CMV. Our results indicate that the correlations between constructs in both groups are indeed below 0.9, strengthening the argument that CMV did not pose a significant issue in this study.

## 4 Results

The analysis of data and estimation of the structural model were carried out utilizing the SmartPLS 4 software. To guarantee the stability and precision of the parameters, the PLS algorithm underwent 300 iterations, and 5,000 bootstrap subsamples were generated, with a 95% confidence interval and a significance level set at 5%, utilizing a two-tailed test. These choices align with recommendations by Hair et al. (2012) and Ringle et al. (2022).

The proposed structural model underwent evaluation using PLS-SEM to assess its fit parameters. In order to determine the model’s acceptability, the Normed Fit Index (NFI) was employed with a threshold of 0.8 or higher, and the Standardized Root Mean Square Residual (SRMR) was used with a threshold of 0.08 or lower (Gupta and Singharia, 2021). The analysis results indicate a favorable fit for the model, as both fitness indices surpassed the threshold values. Specifically, the NFI obtained a value of 0.81, and the SRMR yielded a value of 0.062.

### 4.1 The measurement model

To evaluate the reliability and validity of the measurement model, two criteria were utilized: reliability and validity. To assess reliability, we calculated Cronbach’s alpha and composite reliability (CR) as measures of internal consistency, along with evaluating the outer loadings for each individual item. Table 2 provides descriptive statistics for the indicator items and factor loadings for each item. Notably, all indicator items exhibited outer loadings surpassing the recommended threshold of 0.70 (Fornell and Larcker, 1981), thus signifying satisfactory reliability.

Table 3 outlines the Cronbach’s alpha and composite reliability (CR) values for each construct. The analysis revealed that all constructs had Cronbach’s alpha values exceeding the recommended threshold of 0.70, as suggested by Hair et al. (2012). Additionally, the CR values for all constructs surpassed the established cutoff of 0.70 (Hair et al., 2012).

**Table 3:** Assessment of reliability and convergent validity.

Constructs	Cronbach α	CR	AVE
Academic achievement	0.82	0.88	0.65
Imagination	0.87	0.92	0.79
Immersion	0.90	0.94	0.83
Information quality	0.90	0.94	0.84
Information richness	0.80	0.88	0.71
Learning motivation	0.92	0.94	0.81
Wearable comfort	0.79	0.88	0.71

To assess convergent validity, the study adhered to two key criteria: evaluating the outer loadings of individual items and calculating the average variance extracted (AVE) for each construct (Fornell and Larcker, 1981). The analysis revealed that all items had factor loadings exceeding the recommended threshold of 0.70. Furthermore, the AVE values for all constructs surpassed the cut-off threshold of 0.5 (Fornell and Larcker, 1981). The results from Tables 2, 3 demonstrate satisfactory fit indices for convergent validity, indicating an adequate level of validity.

To assess discriminant validity, three distinct criteria were utilized: (1) an analysis of inter-item cross loadings, (2) the Fornell-Larcker criterion, and (3) the Heterotrait-Monotrait ratio (HTMT) criterion. These criteria were employed to ensure that the constructs in the study exhibited adequate discriminability. As indicated in Table 4, the results reveal that the indicator items measuring each construct exhibit significant and robust correlations among themselves, while also exhibiting higher loadings on their respective constructs. This serves as evidence that supports the discriminant validity of the measurement model.

**Table 4:** Discriminant validity: inter-item cross loading.

Items	Academic achievement	Imagination	Immersion	Information quality	Information richness	Learning motivation	Wearable comfort
AA1	**0.83**	0.47	0.47	0.46	0.57	0.62	0.30
AA2	**0.86**	0.51	0.45	0.44	0.52	0.61	0.31
AA3	**0.82**	0.47	0.39	0.45	0.51	0.59	0.34
AA4	**0.72**	0.50	0.45	0.79	0.51	0.49	0.27
IMGN1	0.58	**0.91**	0.49	0.59	0.52	0.66	0.36
IMGN2	0.50	**0.86**	0.43	0.49	0.48	0.60	0.40
IMGN3	0.55	**0.90**	0.46	0.50	0.51	0.69	0.41
IMRN1	0.48	0.41	**0.88**	0.48	0.46	0.38	0.15
IMRN2	0.50	0.49	**0.94**	0.52	0.48	0.45	0.19
IMRN3	0.52	0.51	**0.92**	0.53	0.52	0.47	0.21
IQ1	0.56	0.53	0.54	**0.91**	0.48	0.47	0.28
IQ2	0.65	0.55	0.51	**0.92**	0.54	0.49	0.27
IQ3	0.63	0.56	0.50	**0.92**	0.51	0.50	0.26
IR1	0.55	0.45	0.47	0.47	**0.86**	0.46	0.30
IR2	0.55	0.47	0.49	0.44	**0.83**	0.49	0.26
IR3	0.56	0.52	0.40	0.50	**0.85**	0.53	0.39
LM1	0.61	0.63	0.37	0.37	0.49	**0.90**	0.39
LM2	0.67	0.71	0.47	0.55	0.58	**0.90**	0.34
LM3	0.65	0.63	0.40	0.47	0.52	**0.91**	0.36
LM4	0.64	0.66	0.47	0.52	0.51	**0.89**	0.38
WC1	0.29	0.32	0.17	0.20	0.28	0.31	**0.84**
WC2	0.32	0.41	0.16	0.27	0.30	0.35	**0.83**
WC3	0.35	0.37	0.18	0.27	0.36	0.37	**0.86**

The bold values represent the highest cross-loadings for each item, indicating the strongest correlation between that particular item and the corresponding construct (i.e., the row heading).

Table 5 displays the Fornell-Larcker correlation matrix, which was employed to evaluate discriminant validity. The diagonal elements represent the square roots of the AVE, while the off-diagonal elements represent the estimated correlations between the respective pairs of constructs (as indicated by the rows and columns). Discriminant validity is deemed satisfactory when the square root of the AVE for each construct is consistently higher than the corresponding inter-construct correlation (Fornell and Larcker, 1981). The results in Table 5 confirm that all square roots of the AVE values exceed their respective squared correlations, thereby validating the satisfactory discriminant validity of the measurement model.

**Table 5:** Discriminant validity: inter-construct correlations (Fornell-Larcker).

Constructs	Academic achievement	Imagination	Immersion	Information quality	Information richness	Learning motivation	Wearable comfort
Academic achievement	0.807						
Imagination	0.609	0.889					
Immersion	0.55	0.518	0.912				
Information quality	0.673	0.595	0.559	0.916			
Information richness	0.655	0.567	0.535	0.557	0.843		
Learning motivation	0.714	0.733	0.475	0.534	0.585	0.898	
Wearable comfort	0.378	0.44	0.201	0.295	0.375	0.408	0.841

The HTMT criterion serves as a reliable metric for assessing discriminant validity by estimating the correlations between distinct constructs. As shown in Table 6, all HTMT values are well below the recommended threshold of 0.90, as advocated by Fassott et al. (2016), thereby indicating robust discriminant validity.

**Table 6:** Discriminant validity: inter-construct correlations (Heterotrait-monotrait ratio).

Constructs	Academic achievement	Imagination	Immersion	Information quality	Information richness	Learning motivation	Wearable comfort
Academic achievement							
Imagination	0.719						
Immersion	0.637	0.583					
Information quality	0.768	0.671	0.621				
Information richness	0.808	0.681	0.634	0.655			
Learning motivation	0.821	0.819	0.518	0.582	0.681		
Wearable comfort	0.466	0.526	0.237	0.344	0.467	0.475	

The thorough evaluation presented above offers compelling evidence that underscores the reliability and validity of the proposed model, encompassing both convergent and discriminant aspects of validity.

### 4.2 The structural model

**Figure 3:**
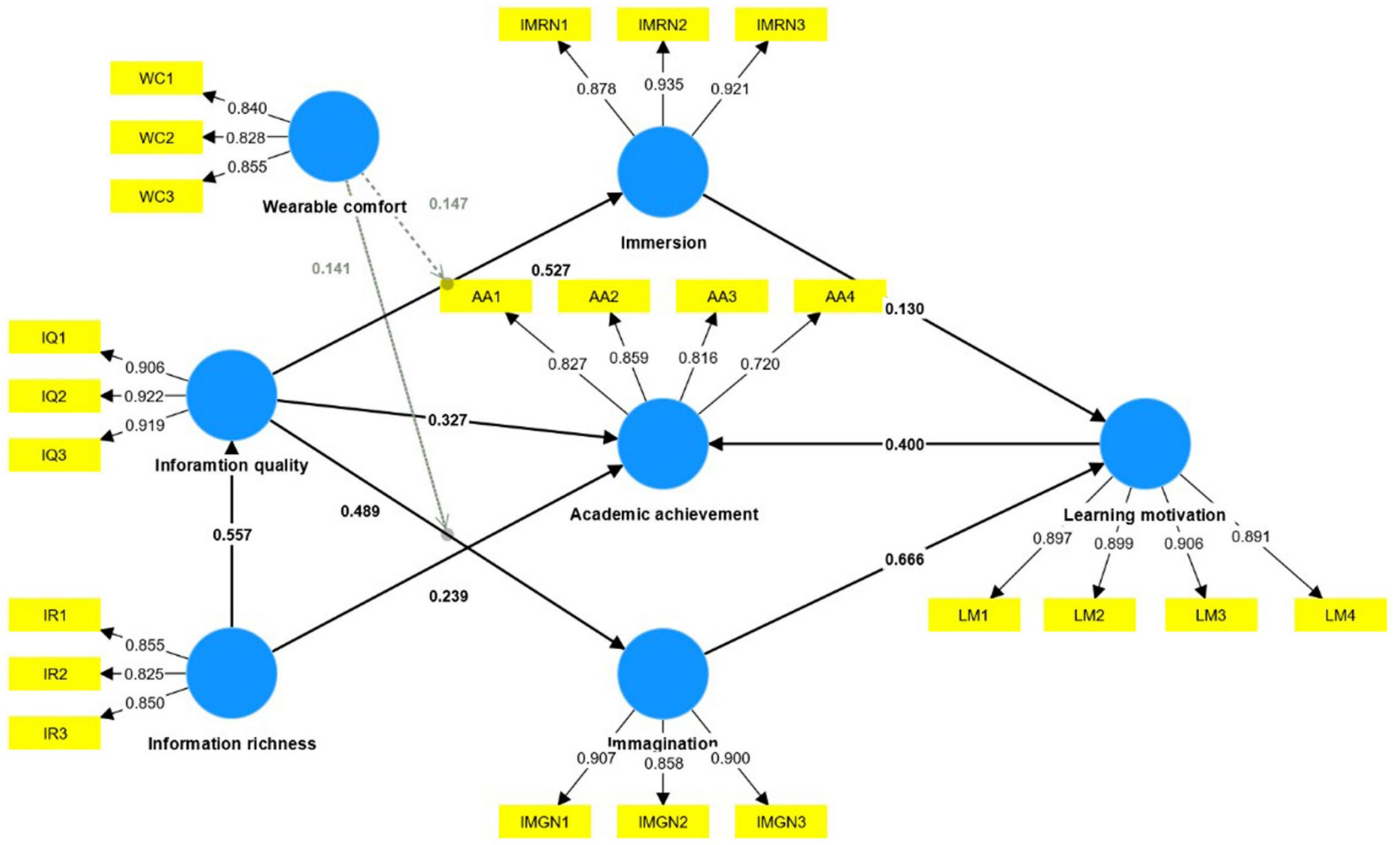
Results of the PLS structural model.

Figure 3 visualizes the proposed structural model, depicting the estimated regression path coefficients (*β*) and the outer loadings of the indicator items, accompanied by their respective significance levels. The figure also incorporates the moderating effect within the research model. As expected, the information richness has a significant effect on information quality (*β* = 0.557, *p* < 0.001) and on academic achievement (*β* = 0.239, *p* < 0.001). Information quality has a significant influence on immersion (*β* = 0.527, *p* < 0.001), on imagination (*β* = 0.489, *p* < 0.01), and on academic achievement (*β* = 0.327, *p* < 0.01). Immersion (*β* = 0.130, *p* = 0.003) and Imagination (*β* = 0.666, *p* < 0.001) has a significant influence on learning motivation. Learning motivation has a significant influence on academic achievement (*β* = 0.400, *p* < 0.001). Wearable comfort has positive moderating effects on the relationship between information quality and immersion (*β* = 0.147, *p* = 0.002), information quality and imagination (*β* = 0.141, *p* = 0.014).

## 5 Discussion and conclusion

Firstly, this study demonstrated significant positive effects of information quality on immersion, imagination, and academic achievement. Additionally, significant positive effects of information richness on academic achievement were also observed, supporting hypotheses 1–4. The information quality of AR can play a crucial role in immersing customers in the destination and stimulating their imagination during the trip. Similar findings were also reported by Lee et al. (2020) in the context of VR implementation in museums, where it was found that the content quality of VR positively influences customers’ telepresence and attitudes toward VR. Consistent with prior research by Kim and Lee (2021), our study also found that the quality of online education significantly impacts academic achievement, with the class content factor exerting the most substantial influence on both satisfaction and academic performance. Several studies have emphasized the significance of information richness in the respective educational environment. Morgan’s study (Shepherd and Martz, 2006) revealed a positive relationship between media richness in a distance education environment and various outcomes. More specifically, it revealed that as the media richness was enhanced, there was a corresponding rise in satisfaction levels with the distance course/program. This augmentation also led to improved communication between students and faculty, as well as a heightened valuation of the course delivery platform by its users. Shaw et al. (2009) argue that online media with high information richness is more effective in facilitating the learning process, particularly in terms of progressing from perception to comprehension.

Secondly, the findings of the study also indicate that information quality plays a mediating role in the relationship between information richness and the constructs of immersion and imagination, thereby providing support for hypothesis 5. According to Zhao et al. (2017) research, individuals who were presented with rich media had notably higher perceptions regarding the quality of information when compared to those who were shown lean media. Jiang et al. (2022) study on the factors that influence the continued usage of AR in museums also yielded similar results, according to which, information richness had a positive impact on the information quality perceived by the users. Zhu et al. (2020) conducted a study exploring the variations in the perceived quality of online reviews, taking into account the information richness theory, emotional polarity, and different types of products. The results indicated a significant difference in the perceived information quality among users who had access to high, medium, and low information richness.

Thirdly, the study revealed that immersion and imagination play a mediating role in the association between information quality and learning motivation, thereby providing support for hypothesis 6 and hypothesis 7. Immersion and imagination, often regarded as inherent characteristics of virtual environments (Mulders et al., 2020), signify the multi-dimensional key attributes of virtual systems and tools that significantly impact system users. Barrett et al. (2021) delved into the extent to which users embraced a high-immersion virtual learning environment specifically tailored for the purpose of mastering the intricacies of English paragraph writing structure. The study found that immersion and imagination had a significant impact on users’ perceived usefulness, which, in turn, influenced their behavioral intention to use the system. The primary value of AR lies in its ability to seamlessly integrate digital elements into a person’s perception of the real world, going beyond mere data display. AR achieves this by incorporating immersive sensations that feel like natural components of the environment. This immersive experience, coupled with the imaginative aspect of AR, has been found to positively impact learning motivation in our study. That might be because the combination of immersion and imagination in AR enhances users’ engagement and interest, leading to increased motivation to learn and explore. Therefore, AR’s ability to blend the digital and physical worlds, along with its immersive and imaginative nature, contributes to its positive influence on learning motivation.

Fourthly, regarding the structural model of AR employed in museums, there exists a positive correlation between learning motivation and academic achievement. This indicates that as users’ motivation intensifies, they are likely to attain higher levels of academic success, thereby validating hypothesis 8. As indicated by previous studies, there is a significant correlation between learning motivation and academic achievement (Khalaila, 2015; Lemos and Veríssimo, 2014). Learning motivation plays a beneficial and constructive role in enhancing academic performance (Su and Cheng, 2019).

Fifthly, the wearable comfort moderates the effect of information quality on immersion and imagination was found in our model, supporting hypothesis 9 and hypothesis 10. One explanation for this phenomenon is that poor wearable comfort can act as a distractor, diverting users’ attention away from the content. Distractions resulting from discomfort can lead to decreased cognitive processing and information retention. In the case of AR, where users are required to process both real-world stimuli and digitally augmented information simultaneously, any additional cognitive load imposed by discomfort can hamper their ability to effectively process and integrate the information provided by the AR system. When users experience discomfort or inconvenience while wearing AR devices, their cognitive resources may be allocated toward addressing the discomfort rather than fully engaging with the AR content. As a result, their ability to become fully immersed in the augmented environment and utilize their imagination to interact with digital elements may be compromised. Lee et al. (2019) proposed that ensuring a comfortable experience in the use of AR and VR technologies is crucial during the development of such technologies. To mitigate the potential negative effects of poor wearable comfort, it is essential for designers and developers to prioritize ergonomic considerations in the design of AR devices. Factors such as weight distribution, adjustability, padding, and ventilation should be carefully addressed to ensure that users can wear the devices comfortably for extended periods without experiencing distractions or discomfort.

Theoretically, this paper highlights the underlying mechanisms through which these variables influence each other and contribute to learning motivation and academic achievement. By identifying these mediating pathways, the study offers insights into the complex interplay between information quality, immersion, imagination, learning motivation, and academic achievement, contributing to the theoretical understanding of the underlying processes in virtual learning environments. These findings expand upon existing literature by highlighting the significance of these factors in enhancing user experiences and outcomes in virtual learning environments.

Practically, the findings of this study have significant implications for the design and implementation of AR-based learning systems and applications. Designers and developers should strive to provide high-quality and rich information to users, as these factors significantly influence users’ immersion, imagination, and academic achievement. The study emphasizes the role of immersion and imagination in promoting learning motivation. Educators and instructional designers can leverage these factors to enhance learner engagement and interest. By incorporating immersive and imaginative elements into AR experiences, such as interactive simulations, gamification, and storytelling, the learning environment can be enriched, fostering a sense of curiosity, exploration, and active participation. The study also highlights the importance of wearable comfort in AR experiences. Developers and manufacturers of AR devices should prioritize ergonomic design principles to ensure user comfort and minimize distractions. By addressing issues related to weight, adjustability, padding, and ventilation, AR devices can be made more user-friendly and conducive to prolonged use.

## 6 Limitations and future research

Despite the valuable insights garnered through this research, there are still some limitations that require further investigation. Firstly, it is important to acknowledge that our study focused on a sample of tourists visiting a specific museum in China. However, the generalizability of the findings to museum tourists from diverse countries or regions is yet to be determined. Therefore, future research endeavors should aim to collect data from museum tourists representing different countries or regions to enhance the external validity and robustness of these findings. Secondly, it is worth noting that the current study specifically examined a natural history museum as the experimental setting. While the findings provide valuable insights into the relationship between variables within this context, it is important to extend the research to other types of museums. Future studies could explore and compare the applicability and effectiveness of the model in different museum settings, such as art museums, science museums, or cultural heritage museums. This would lead to a more comprehensive understanding of the model’s generalizability and applicability across various museum contexts, enriching our knowledge in the field. Additionally, our study employed a specific model of AR glasses and a particular AR content design, which may limit the broader applicability of our findings. Different AR glasses with varied technical features or affordances—such as higher resolution, broader field of view, or enhanced interactive capabilities—could yield different results. Similarly, an AR environment offering more interactive experiences between users and virtual objects might influence immersion, imagination, and other key outcomes differently. Therefore, future research should explore alternative AR devices and more interactive content designs to assess whether these findings hold across diverse technological contexts and user experiences.

## Data Availability

The raw data supporting the conclusions of this article will be made available by the authors, without undue reservation.
